# We need leaders that believe in scientific evidence

**DOI:** 10.1371/journal.pbio.3000992

**Published:** 2020-10-22

**Authors:** Nonia Pariente, on behalf of the PLOS Biology Staff Editors

**Affiliations:** Public Library of Science, San Francisco, California, United States of America and Cambridge, United Kingdom

## Abstract

In a world beset by attempts to undermine scientific evidence and evidence-based policy, we emphasize their important role in helping humanity rise to the challenges of our time.

The scientific enterprise has great potential to benefit all aspects of society, as well as to increase our understanding of the world and how it is changing in response to our actions. The advances brought about by research are too many to enumerate; one only needs to think of our successes in identifying the causes of disease and developing appropriate treatments, or the myriad technological advances that are part of our daily lives. For research to serve its purpose of benefitting society, however, it needs to engender something that is as important as it is fragile—trust. Trust in science is essential for it to effectively inform policy and more fully benefit society.

But this trust is increasingly being undermined across the world by those elected to make decisions in the name of and for the benefit of society. *PLOS Biology*, founded on the principle of accelerating progress in science, cannot stand by in silence.

Both public and political leaders once embraced great advances, such as the polio vaccine, and worrying truths, such as the evidence that our use of chlorofluorocarbons (CFCs) was destroying the ozone layer. As a result, mass vaccination campaigns have all but eradicated polio and the Montreal protocol, a global agreement to phase out ozone-depleting substances, saved the ozone layer. Today, many people reject the possibility of using a coronavirus vaccine even as scientists race to develop one, the US President has pulled out of the World Health Organization (WHO) and the Paris Agreement even as the world burns around us, and the destruction of the Amazon has hit a record high under the current Brazilian Administration. Across the world we are witnessing attacks on scientific independence and the undermining of institutions that have been historical pillars of evidence-based policy, such as leading centers for the control of disease and those that protect the environment and regulate medicine approval. We are also witnessing flagrant misinformation and denial of scientific evidence from many leaders who should be acting in the best interest of their population, even when it concerns the most basic of viral transmission control measures or when confronted with satellite images evidencing the truth. Treatments that are the result of many years of research and development are touted as miracles, undermining the role of scientists in bringing them to the clinic in favour of more demagogic interpretations. Withdrawal from essential international organizations and agreements, and even the gagging or discrediting of individual researchers for political purposes—things that would have been unthinkable a few years ago—have come to pass.

The independence of scientific research, a crucial tenet on which scientific advance is based, is important to avoid politization of work that is for the most part funded by public money. For science to maximally benefit society and inform policy, research should be conducted in an unbiased manner, and results should be objectively and freely communicated, independently of the agenda of the body funding the research. Likewise, decisions on science funding and support should not be driven by political or personal beliefs.

In these times of social media echo-chambers, disinformation and fake news, leaders would be well advised to use their voices to bolster rather than to undermine trust in science, expertise and evidence-based decision-making. Our societies need voices that point to a way forward in the road ahead—with its uncertainties, certainly, but based on the most solid scientific advice available—rather than contribute to the chaos and polarization. There are excellent examples of evidence-based decision-making in response to the COVID-19 pandemic, and these countries are for the most part controlling it with limited disruption to normal life, whereas chaotic responses and mounting death tolls plague those ignoring the scientific advice. We live in extraordinary times, in which belief in the existence of an infectious agent, or that our planet is warming, seems to depend more on one’s general political ideology than on rational arguments.

This editorial could single individuals out by name but the sad truth is that these issues are pervasive throughout the world and at all levels of government. Shining a spotlight in any one place would take it away from others. We are confident that our readers will be able to fill in the gaps relevant to their particular circumstances.

We are in the middle of a pandemic and are destroying the one planet we have to live on. Science can help us find ways to protect ourselves from the former and protect the latter from us. This, however, requires that we as a global population embrace evidence-based decision-making and trust our experts to be objectively seeking possible solutions. Those of us that have the good fortune of living in democratic societies will have a chance to make our voices heard. We urge you to take advantage of that opportunity, vote and have these issues uppermost in your mind when casting your ballot. Our votes will decide who the next world leaders will be and where they will take us.

